# Sarcopenia and Mediastinal Adipose Tissue as a Prognostic Marker for Short- and Long-Term Outcomes after Primary Surgical Treatment for Lung Cancer

**DOI:** 10.3390/cancers15235666

**Published:** 2023-11-30

**Authors:** Florian Ponholzer, Georg Groemer, Caecilia Ng, Herbert Maier, Paolo Lucciarini, Florian Kocher, Dietmar Öfner, Eva Gassner, Stefan Schneeberger, Florian Augustin

**Affiliations:** 1 Department of Visceral, Transplant and Thoracic Surgery, Center of Operative Medicine, Medical University of Innsbruck, 6020 Innsbruck, Austria; florian.ponholzer@i-med.ac.at (F.P.); georg.groemer@student.uibk.ac.at (G.G.); caecilia.augustin@tirol-kliniken.at (C.N.); dietmar.oefner@i-med.ac.at (D.Ö.);; 2 Department of Internal Medicine V: Hematology and Oncology, Medical University Innsbruck, 6020 Innsbruck, Austria; 3 Department of Radiology, Medical University Innsbruck, 6020 Innsbruck, Austria

**Keywords:** sarcopenia, lung cancer, VATS, morbidity, mediastinal adipose tissue

## Abstract

**Simple Summary:**

Surgical resection is the current gold standard of treatment for early-stage lung cancer. With the rising age and number of patients, prognostic factors for morbidity and oncological outcomes have to be taken into consideration to set expectations within reason and to identify those who benefit most from surgical treatment. The aim of this study was to explore the impact of preoperative sarcopenia and mediastinal adipose tissue on postoperative and oncological outcomes after primary surgical treatment for lung cancer. In total, 311 patients undergoing lobectomy or segmentectomy were analysed. Our study showed that sarcopenia is an independent risk factor for postoperative morbidity and impaired five-year overall survival. Mediastinal adipose tissue showed no association with postoperative outcomes in our study.

**Abstract:**

Surgical resection remains the gold standard of treatment for early-stage lung cancer. Several risk models exist to predict postoperative morbidity and mortality. Psoas muscle sarcopenia has already successfully been used for morbidity prediction in lung transplantation and is not yet included in the available risk scores for pulmonary resections. We hypothesized that the skeletal muscle index and mediastinal adipose tissue might also have an impact on postoperative outcomes after primary surgery for primary lung cancer. The institutional database was queried for patients with primary lung cancer who were treated with primary lobectomy or segmentectomy between February 2009 and November 2018. In total, 311 patients were included for analysis. Patients receiving neo-/adjuvant chemotherapy or with a positive nodal status were excluded to rule out any morbidity or mortality due to (neo-)adjuvant treatment. Sarcopenia was defined as a skeletal muscle index of <34.4 cm^2^/m^2^ for women and <45.4 cm^2^/m^2^ for men. Mediastinal adipose tissue was defined with a radiodensity of −150 to −30 Hounsfield units. Sarcopenia was diagnosed in 78 (25.1%) of the 311 patients. Male patients were significantly more likely to suffer from sarcopenia (31.5% vs. 18.1%, *p* = 0.009). Comorbidities, lung function, tumour histology, pathologic tumour staging, mediastinal adipose tissue and age did not differ between groups with or without sarcopenia. Sarcopenic patients had a significantly longer length of stay, with 13.0 days vs. 9.5 (*p* = 0.003), and a higher rate of any postoperative complications (59.0% vs. 44.6%, *p* = 0.036). There was no difference in recurrence rate. Five-year overall survival was significantly better in the patient cohort without sarcopenia (75.6% vs. 64.5%, *p* = 0.044). Mediastinal adipose tissue showed no significant impact on length of stay, postoperative complications, recurrence rate, morbidity or survival. Sarcopenia, quantified with the skeletal muscle index, is shown to be a risk factor for postoperative morbidity and reduced survival in primary lung cancer. Efforts should be taken to pre-emptively screen for sarcopenia and start countermeasures (e.g., physical prehabilitation, protein-rich nutrition, etc.) during the preoperative workup phase.

## 1. Introduction

With rising numbers of patients diagnosed with early-stage lung cancer and various equally accepted treatment modalities with comparable outcomes, there is an ongoing discussion on which patient groups should be directed to which treatment modality. A recent meta-analysis, comparing stereotactic ablative radiotherapy and surgery in early-stage lung cancer, showed favourable results of surgery with significantly prolonged survival [1]. Contrary to this analysis, the prospective STARS trial reported the non-inferiority of stereotactic ablative radiotherapy in comparison to video-assisted thoracoscopic surgical lobectomy with mediastinal lymph node dissection. Nevertheless, the surgical cohort showed a significantly lower regional recurrence rate of 2.7 vs. 12.5% (*p* = 0.02) [2]. Until now, different risk scores, like EuroLung scores, have tried to stratify the risk for postoperative morbidity and mortality for each patient, but our data have shown that these scores fail to reliably predict patients’ individual risks. This might be due to different patient populations or morbidity-influencing factors not being included, as these factors were not available in the databases used for calculating the risk scores [3]. 

Sarcopenia and psoas muscle index represent an indicator for the functional capacities of patients and their reserves. Furthermore, these factors are naturally associated with the ageing process. Objective measurements of skeletal muscle cross-sectional area can be performed and quantified with computer tomography (CT) [4]. They have already been successfully used for morbidity prediction in liver transplantation and cardiovascular surgery. In thoracic surgery, the psoas muscle index was assessed for oncologic outcomes regarding squamous cell carcinoma and adenocarcinoma [5,6,7,8]. Moreover, mediastinal adipose tissue (MAT) has been shown to be a prognostic marker for metabolic syndrome, morbidity and mortality in some selected patient cohorts. In these studies, higher amounts of MAT correlated with a higher rate of adverse effects [9,10,11,12,13]. Nevertheless, these parameters are not included in the currently available risk scores for pulmonary surgery.

We hypothesized that the psoas muscle index also has an impact on postoperative outcomes after primary surgery for lung cancer based on the available literature. Therefore, the aim of this study is to examine the value of a CT-measured psoas muscle index in postoperative morbidity assessment. Additionally, mediastinal adipose tissue as a possible marker of sarcopenia is measured and assessed for its impact. Furthermore, this study examines if there is a connection between sarcopenia according to the height-adjusted skeletal muscle index (SMI) and MAT, as a metabolic marker, because there is no sufficient data available in the literature.

## 2. Materials and Methods

### 2.1. Patient Selection

All patients with primary lung cancer, who were treated via primary video-assisted thoracoscopic surgery between February 2009 and November 2018, were retrospectively analysed. Patients who received primary thoracotomy, pneumonectomy or neo-/adjuvant chemotherapy were excluded from analysis. Further exclusion criteria, to eliminate possible confounders, were as follows: positive pathologic lymph node staging, insufficient radiological imaging or patient data. The resulting patient cohort should be as homogeneous as possible to rule out any resulting morbidity or mortality due to (neo-)adjuvant treatment. In total, 311 patients were left for analysis. Permission for analysis was granted by the local ethics committee (registration number: AN5163 327/4.17 382/5.2).

### 2.2. Data Collection

Patient data were collected in a prospectively maintained database and included, but were not limited to the following: age, sex, body mass index (BMI), lung function parameters, parameters measuring sarcopenia, comorbidities, length of stay, type of operation, morbidity, mortality, disease-free survival and overall survival.

**Figure 1:**
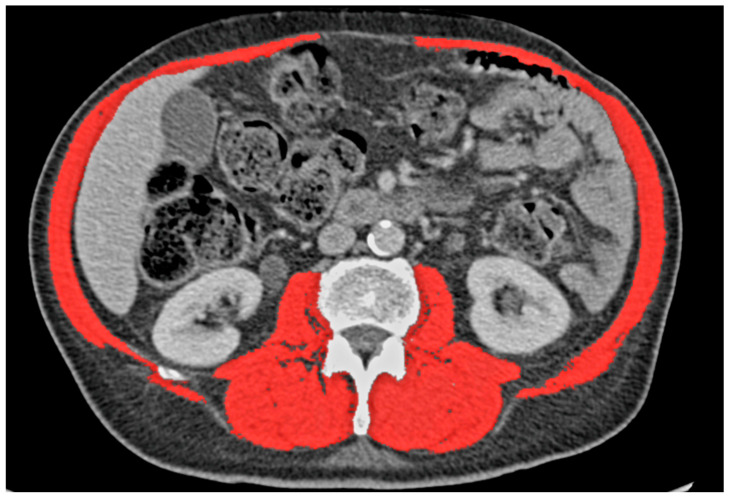
Marked skeletal muscle.

For the measurement of the skeletal muscle cross-sectional area (SMCA) and MAT, the most recent preoperative CT scan was used (with a cut-off of 6 months before surgery). Measurement was performed with OsiriX Lite (v11.0, Pixmeo, Geneva, Switzerland) at the level of the upper border of the third lumbar vertebra (L3). L3 was used, because at this level, SMCA has shown the highest values [4]. The localization of L3 was reached with coronal and sagittal multiplanar reformats [14]. The measurement of SMCA was performed using a semi-automated method, as described by Volbers et al. for perihemorrhagic edema, utilizing the OsiriX “grow region (2D/3D segmentation)” tool for region growing [15]. After selecting a starting point in the region of interest, the software calculated the corresponding area. Skeletal muscle mass was defined with a Hounsfield unit (HU) ranging from −29 to 150 HU, as was described by Derstine et al. [16]. Nevertheless, the investigators of this study performed controls of all marked areas and removed non-skeletal muscle tissue from the measurement, as can be seen in Figure 1.

**Figure 2:**
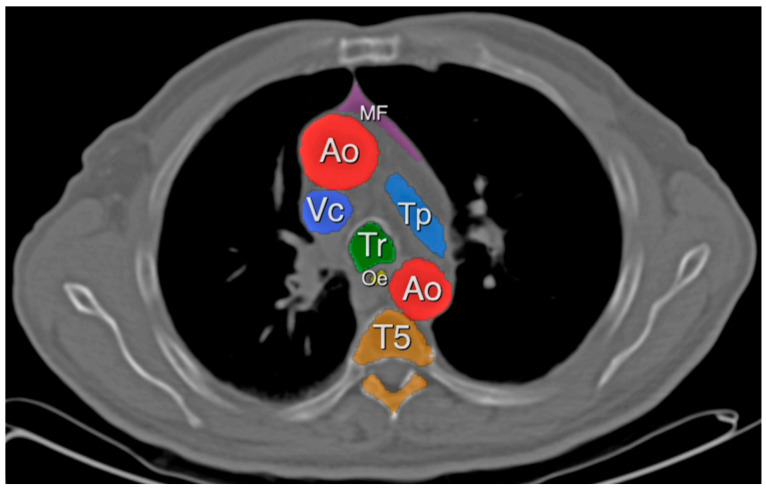
Marked mediastinal adipose tissue (MF). Aorta (Ao), caval vein (Vc), pulmonary trunk (Tp), trachea (Tr), oesophagus (Oe) and fifth thoracic vertebra (T5).

For the quantification of MAT, the same method was used with a HU ranging from −150 to −30, as was described by Marchiori et al. (Figure 2). Measurements for MAT were taken at the height of the tracheal carina [17].

### 2.3. Definitions

Sarcopenia in our study is defined as an SMI of <34.4 cm^2^/m^2^ in women and <45.4 cm^2^/m^2^ in men [4]. These values are in line with the recommendations of the European Working Group on Sarcopenia in Older People (EWGSOP) on using a cut-off value of two standard deviations (SD) below the mean values of healthy adults (women: 47.5 cm^2^/m^2^; men: 60.9 cm^2^/m^2^) [4,18]. 

For the analysis of MAT, patients were assigned to three groups:

Low MAT (cut-off two SD below mean): <1.40 cm^2^/m^2^ for women and <3.70 cm^2^/m^2^ for men;

Medium MAT (mean +/− two SD): 1.40–8.95 cm^2^/m^2^ for women and 3.70–12.80 cm^2^/m^2^ for men;

High MAT (cut-off two SD above mean): >8.95 cm^2^/m^2^ for women and >12.80 cm^2^/m^2^ for men.

We then compared the high-MAT group to the group with normal or low MAT to look for effects on postoperative outcomes.

Complications were graded in accordance with the Clavien–Dindo classification [19] and split into minor (Clavien–Dindo ≤ 2) and major (Clavien–Dindo > 2) complications.

Patients’ comorbidities were graded according to the age-adjusted Charlson comorbidity index (aCCI) [20].

### 2.4. Statistical Analysis

Statistical analysis was performed using IBM SPSS Statistics 26 (IBM Corporation, Armonk, NY, USA) and the R software environment (Version 4.3.1) with the addition of the ‘survminer’ package for plotting survival curves [21,22]. Pearson’s chi-squared test or Fisher’s exact test was used for identifying relationships between categorical variables. For the analysis of means, a t-test was performed as this test is proven to show robustness even if the assumption of normality is violated [23,24,25]. To assess the significance of sarcopenia, binomial logistic regression was performed. Beforehand, the linearity of the variables was checked using the Box–Tidwell method [26]. For an analysis of confounders for postoperative morbidity and five-year overall survival, variables used in the Eurolung1 and Eurolung2 models by Brunelli et al. were included in binomial logistic regression and Cox regression [27]. Regarding this matter, the variables from the full model were chosen to include more possible confounders and because of their better calibration and correlation [3]. Statistical significance was assumed for *p*-values < 0.05.

## 3. Results

### 3.1. Skeletal Muscle Index

In total, 311 patients were analysed. The mean age of the study population was 64.66 years (SD 10.65), with 149 (47.9%) female patients. Further patient characteristics are shown in Table 1. Sarcopenia was diagnosed in 78 patients (25.1%).

The mean SMCA was 137.68 cm^2^ in the non-sarcopenic group in comparison to that of 112.98 cm^2^ in the sarcopenic group (*p* < 0.001). The mean SMI differed, with 48.37 and 37.01 cm^2^/m^2^ (*p* < 0.001). Naturally, height, weight and BMI were also significantly different between groups (168.2 vs. 174.00 cm, *p* < 0.001; 74.68 vs. 70.50 kg, *p* = 0.032; 26.30 vs. 23.14, *p* < 0.001). No difference in comorbidities was found between groups. Furthermore, forced expiratory volume in one second in % (FEV1%) and predicted postoperative FEV1 in % (ppoFEV1%) were comparable. Although MAT was hypothesized to be a possible predictor of sarcopenia, the MAT amount was not significantly different between groups. Interestingly, the tumour diameter was significantly smaller in the sarcopenic cohort (18.95 vs. 22.35 mm, *p* = 0.005).

Regarding patient outcomes, sarcopenia was associated with higher rates of postoperative minor and major complications, at 38.5% and 20.5% (vs. 34.8% and 9.9%, *p* = 0.023). Performing binomial logistic regression using variables from Brunelli et al.’s Eurolung1 model and sarcopenia, only sarcopenia and ppoFEV1% remained significant in the multivariate analysis, as shown in Table 2. Patients with sarcopenia also had a significantly higher Eurolung1 probability for postoperative morbidity, with 11.66 vs. 10.47% (*p* = 0.043) [27].

Length of stay was significantly shorter in the non-sarcopenic cohort, at 9.52 vs. 13.03 days (*p* = 0.003). 

**Figure 3:**
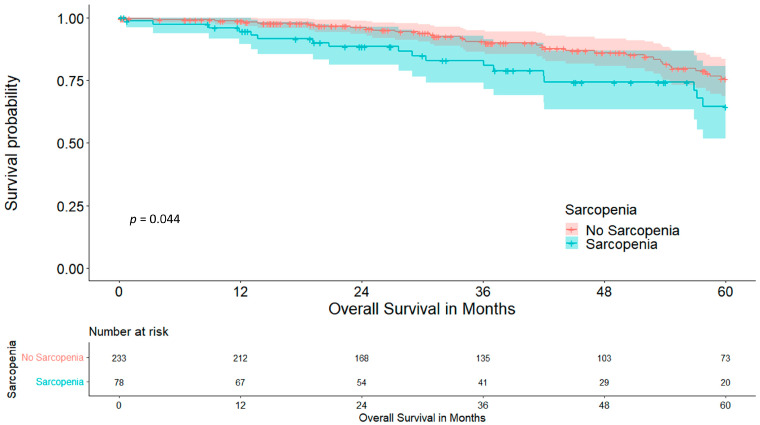
Five-year overall survival stratified for sarcopenia.

Overall five-year survival was significantly higher in the non-sarcopenic cohort, at 75.6 vs. 64.5% (*p* = 0.044), as shown in Figure 3.

Performing Cox regression analysis using variables from Brunelli et al.’s Eurolung2 model and sarcopenia, sarcopenia and extended resection showed a trend towards significance for five-year survival (Table 3). Sarcopenic patients also had a significantly higher Eurolung2 probability for postoperative mortality, at 1.31 vs. 0.90% (*p* < 0.001) [27].

**Figure 4:**
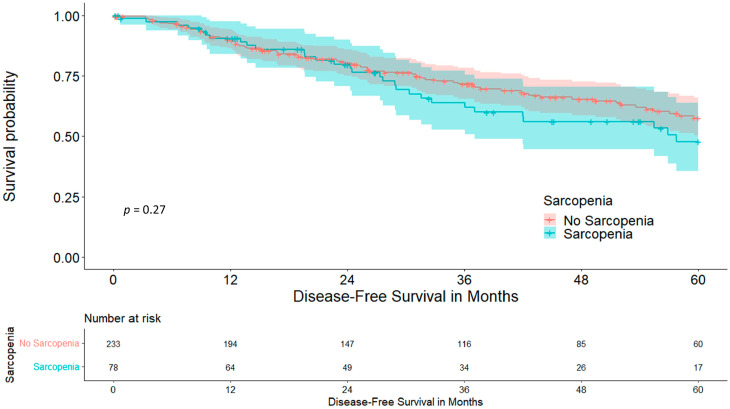
Five-year disease-free survival stratified for sarcopenia.

Disease-free survival did not differ statistically between cohorts, at 57.6 vs. 47.8% (*p* = 0.273), as shown in Figure 4.

### 3.2. Mediastinal Adipose Tissue

High MAT was measured in 54 patients (17.36%). Patients with high MAT were significantly older, at 68.61 vs. 63.83 years (*p* < 0.001). Patient characteristics of the cohort can be seen in Table 4.

The mean SMCA was 129.27 cm^2^ in the low-/medium-MAT group in comparison to 142.02 cm^2^ in the high-MAT group (*p* = 0.005). The mean SMI differed, with 44.80 and 48.94 cm^2^/m^2^ (*p* = 0.002). Furthermore, weight and BMI were significantly different between groups (70.42 vs. 88.88 kg, *p* < 0.001; 24.42 vs. 30.68, *p* < 0.001). Height did not differ between groups. The high-MAT cohort showed a significantly lower rate of COPD, but on the other hand a higher rate of diabetes (37.4 vs. 13.0%, *p* < 0.001; 10.1 vs. 25.9%, *p* = 0.003). No difference in other comorbidities was found between groups. FEV1% and ppoFEV1% were comparable. MAT was not significantly associated with sarcopenia; even when comparing low MAT vs. medium/high MAT, no significant association was seen (33.9 vs. 23.1%, *p* = 0.124).

Regarding patient outcomes, MAT had no significant association with rates of postoperative minor and major complications or length of stay. 

**Figure 5:**
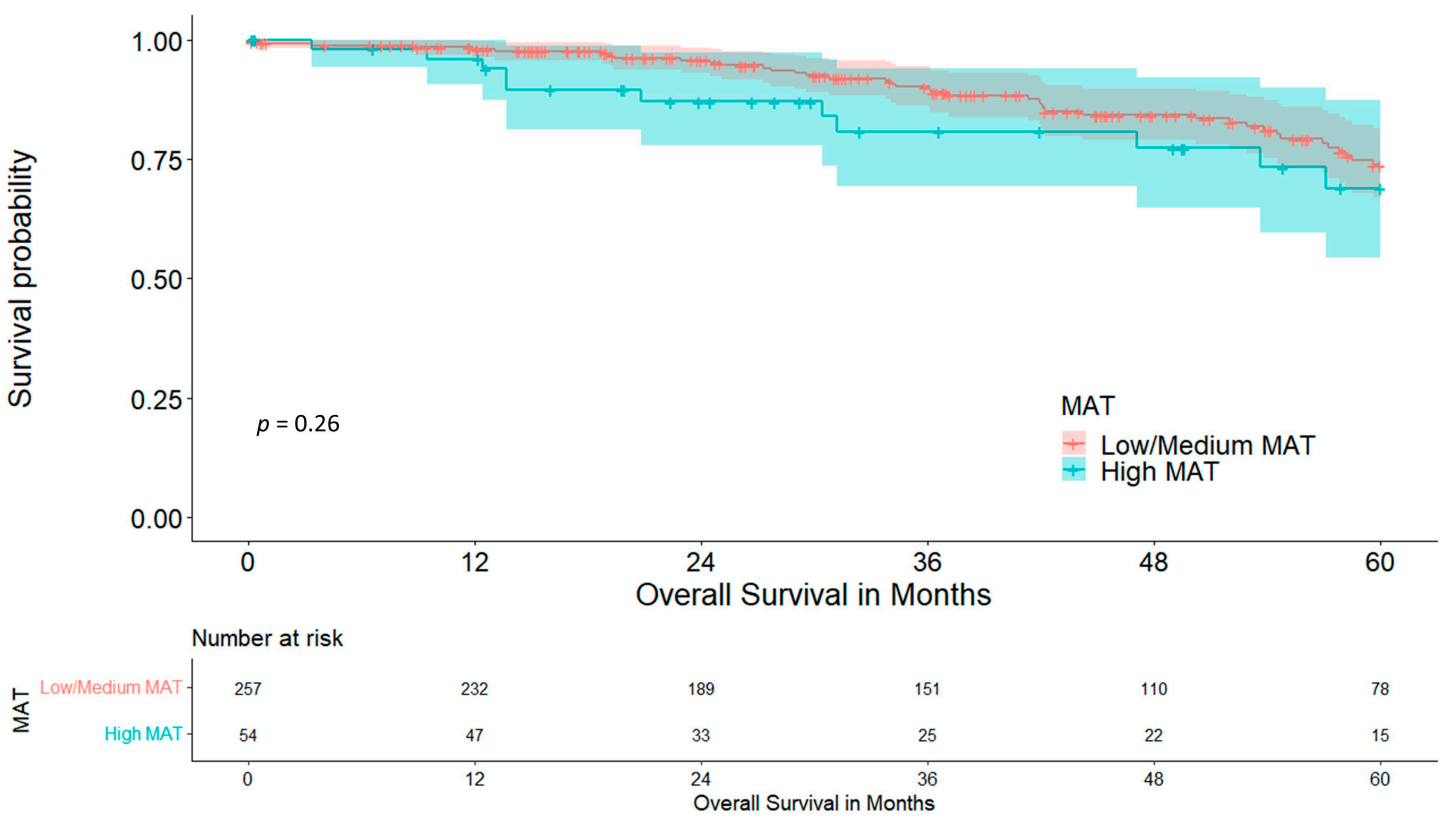
Five-year overall survival stratified for mediastinal adipose tissue.

Although the low-/medium-MAT cohort showed a graphical trend towards longer overall five-year survival, no statistical significance was reached, at 73.8 vs. 68.9% (*p* = 0.261), which is visualized in Figure 5.

**Figure 6:**
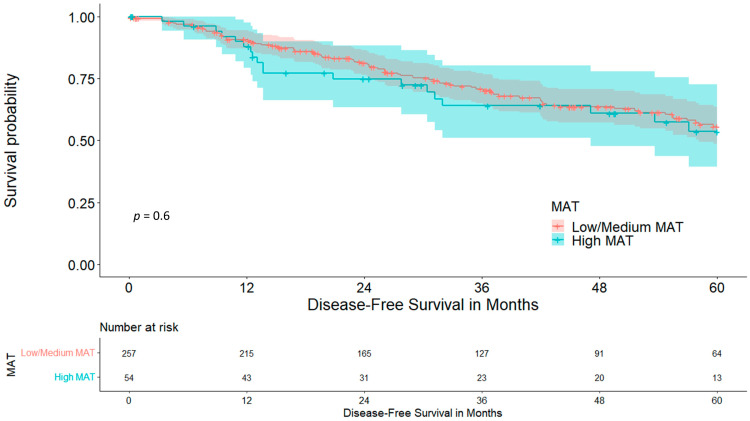
Five-year disease-free survival stratified for mediastinal adipose tissue.

Disease-free survival did not differ between cohorts, at 55.6 vs. 53.6% (*p* = 0.599), which is visualized in Figure 6.

A further analysis of low MAT vs. medium/high MAT showed no significant association with complication rates, overall five-year survival or five-year disease-free survival.

## 4. Discussion

With the rising number of cases of lung cancer detected in early stages, more patients are eligible for curative treatment strategies. Accordingly, it is of importance to have modes of risk stratification, as all available treatment options have their own risk–benefit ratios. Members of the interdisciplinary tumour board have to rely on their experience and, if available, on risk prediction models to recommend an optimal treatment strategy balancing risks and benefits. Nevertheless, these risk prediction models, even if based on large patient cohorts, often only provide poor accuracy, as we previously showed in an external validation of the ESTS EuroLung scores [3]. A likely problem of the mentioned risk scores might be that some possible risk factors were not included in the creation of the models. One of these factors is sarcopenia, which can be defined by the patient’s SMI and should be measurable for any oncological patient as body CT scans are usually available during patient work-up. Likewise, MAT can be routinely measured and quantified. Our study aimed to analyse these two values as possible risk factors for postoperative outcomes, which might be included in future reworked risk scores.

When looking at sarcopenia, both cohorts presented with comparable comorbidities and aCCI, making confounders for postoperative complications rather unlikely. This is essential for this analysis as some of these comorbidities were shown to be risk factors for postoperative morbidity in our cohort [3,28]. Interestingly, sarcopenia and MAT had no association with each other (*p* = 0.117) and seemed to be based on different mechanisms. The amount of patients without postoperative complications was significantly higher in non-sarcopenic patients (55.4 vs. 41.0%, *p* = 0.023). Furthermore, we found a significantly lower amount of major complications (9.9 vs. 20.5%, *p* = 0.023). The exact reason for this result is not yet clear, but might be due to better muscular respiratory function, as diaphragm thickness and its dynamics have proven to be an indicator for postoperative complications and increased respiratory impairment [29]. Other confounders remain unlikely as only sarcopenia and ppoFEV1% remained significant in the multivariate binomial logistic regression analysis. Nevertheless, this hypothesis needs to be further investigated by correlating ultrasound-measured diaphragm thickness with sarcopenia. As a result of these complications the LOS was also longer in the sarcopenic cohort by about three and a half days (9.52 vs. 13.03 days, *p* = 0.003), which puts further economic strain on health care systems [30]. Overall five-year survival was reduced in the sarcopenic cohort, at 64.5% in comparison to 75.6% (*p* = 0.044), while five-year disease-free survival, although it was lower in the sarcopenic cohort, did not significantly differ (57.6 vs. 47.8%, *p* = 0.273). This suggests that not the tumour itself, but sarcopenia and its associated impairments, reduce long-term outcomes. BMI as a possible confounder was ruled out in the Cox regression (*p* = 0.599). These results are further strengthened by the significantly smaller tumour diameter in the sarcopenic cohort (18.95 vs. 22.35 mm, *p* = 0.005), making sarcopenia a risk factor for impaired postoperative outcomes irrespective of tumour size and recurrence. The relevant studies in the literature confirm these data, with reduced survival in sarcopenic patients with liver cirrhosis and various cancers [31,32,33]. One reason for this might be general cancer-associated inflammation as Tsukioka et al. have shown a correlation between sarcopenia and an elevated neutrophil/lymphocyte ratio in non-small-cell lung cancer patients [34]. An elevated neutrophil/lymphocyte ratio on the other hand is associated with reduced overall and disease-free survival in patients with solid tumours [35]. Although sarcopenia is dismal for perioperative outcomes and long-term overall survival, these results are promising, because of the possible preoperative treatment of sarcopenia, which ranges from nutrition and exercise to stem cell therapy, with the first two being easily implementable in the preoperative work-up period [36,37].

Nevertheless, the question of the optimal duration of prehabilitation remains unsolved. A four-week pulmonary exercise program, for instance, was able to achieve a significant reduction in postoperative morbidity and LOS [38]. More recently, a 7-day intensive prehabilitation program including inspiratory muscle training and aerobic endurance training was able to show improved postoperative outcomes even for patients aged 70 years or older who were scheduled for lung cancer surgery [39]. One must not forget the impact of timely surgery in lung cancer treatment, as we have previously shown [40]. Binguel et al., in a recently published review, suggest a two-week schedule with aerobic endurance and inspiratory muscle training together with appropriate nutrition and lifestyle changes for now [41]. Of note is that, while there seems to be a benefit for the overall population, none of the available studies have specifically evaluated the effect of prehabilitation on sarcopenic patients. This needs to be clarified in future studies.

The mediastinum represents a location with a rather high concentration of brown adipose tissue (BAT), which plays an important role in various metabolic pathways and is associated with adipose tissue redistribution during ageing. As the amount of BAT usually decreases with increasing age, it is a surprise that our cohort with high MAT was significantly older than the cohort with low/medium MAT (68.61 vs. 63.83 years, *p* < 0.001) [42,43]. This might be due to a redistribution of white adipose tissue to the mediastinal compartment instead, which is not sufficiently differentiable via a CT scan. As we hypothesized that MAT is associated with the patient’s nutritional status and because of its higher concentration of BAT being responsible for metabolic problems, such as inflammation and insulin resistance, we analysed its impact on postoperative outcomes [43]. The cohort with high MAT had a significantly higher rate of diabetes mellitus, at 25.9% in comparison to 10.1% (*p* = 0.003), which reinforces the hypothesis on adipose tissue redistribution. Interestingly, the rate of COPD was significantly lower within the high-MAT group (13.0 vs. 37.4%, *p* < 0.001). The interpretation of this result remains unclear at this point as the literature regarding BAT activity and distribution in COPD is scarce, although it may be hypothesized that COPD as a hypermetabolic disease requires higher amounts of BAT for homeostasis, which would explain the higher COPD rate in the low-/medium-MAT group. Another indication for this is the fact of a significantly lower BMI in the low-/medium-MAT group (24.42 vs. 30.68, *p* < 0.001), as BAT activity is higher in patients with a lower body fat percentage. [44] The amount of MAT was not associated with the existence of sarcopenia (*p* = 0.124), despite the SMI being lower in the low-/medium-MAT cohort (44.80 vs. 48.94, *p* = 0.002). Moreover, the rate of postoperative complications was comparable between groups. The high-MAT cohort showed a graphical trend towards reduced five-year overall survival, but did not reach statistical significance (73.8 vs. 68.9%, *p* = 0.261); disease-free five-year survival was, as well, comparable (55.6 vs. 53.6%, *p* = 0.599).

## 5. Conclusions

According to our results, sarcopenia should be considered a prognostic marker when assessing the risk of postoperative morbidity and reduced long-term survival. Moreover, sarcopenia needs inclusion in future risk prediction models to better depict the patient’s state of health. An advantage of this consideration is that sarcopenia is potentially treatable, or at least improvable. Nevertheless, for proving a potential benefit through the treatment of sarcopenia, one has to consider the possible delay in treatment for lung cancer, which may also result in dismal outcomes if treatment is initiated too late. MAT provides no value in assessing postoperative morbidity or overall survival in surgically treated lung cancer.

## 6. Limitations

The limitations of this study mainly include its retrospective design. However, we decided to choose rather strict inclusion and exclusion criteria to create a homogeneous patient cohort, whose postoperative course was influenced as little as possible by factors other than sarcopenia. Furthermore, no data on the cause of death are available, which would ultimately demonstrate the impact of sarcopenia on survival.

## Figures and Tables

**Table 1:** Patient characteristics determined via the sarcopenia subanalysis.

Factor	Total (n = 311)	Non-Sarcopenic (n = 233)	Sarcopenic (n = 78)	*p*-Value
Mean Age in years (range)	64.66 (15–83)	64.09 (15–83)	66.38 (36–83)	0.099
Sex (%)				**0.009**
Female	149 (47.9)	122 (52.4)	27 (34.6)	
Male	162 (52.1)	111 (47.6)	51 (65.4)	
Mean BMI (range)	25.5 (14.1–42.3)	26.30 (15.42–42.29)	23.14 (14.13–38.53)	**<0.001**
Mean Height in cm (range)	170 (145–196)	168 (145–196)	174 (152–192)	**<0.001**
Mean Weight in kg (range)	73.63 (38.0–118.0)	74.68 (43–117)	70.50 (38–118)	**0.032**
Mean aCCI (range)	3.13 (0–8)	3.04 (0–8)	3.38 (0–8)	0.102
Mean SMCA in cm^2^ (range)	131.49 (72.93–199.26)	137.68 (83.06–199.26)	112.98 (72.93–167.22)	**<0.001**
Mean SMI in cm^2^/m^2^ (range)	45.52 (26.43–70.22)	48.37 (34.51–70.22)	37.01 (26.43–45.38)	**<0.001**
Mean FEV1% (range)	82.52 (34.0–154.8)	82.45 (34.0–154.8)	82.75 (48.0–135.7)	0.889
Mean ppoFEV1% (range)	65.83 (32.21–130.36)	65.17 (32.21–130.36)	66.02 (37.89–107.13)	0.641
Postoperative Complications (%)				**0.023**
No Complication	161 (51.8)	129 (55.4)	32 (41.0)	
Minor Complication	111 (35.7)	81 (34.8)	30 (38.5)	
Major Complication	39 (12.5)	23 (9.9)	16 (20.5)	
MAT group (%)				0.117
Low MAT	56 (18.0)	37 (15.9)	19 (24.4)	
Medium MAT	201 (64.6)	151 (64.8)	50 (64.1)	
High MAT	54 (17.4)	45 (19.3)	9 (11.5)	
Coronary Artery Disease (%)	14 (4.5)	11 (4.7)	3 (3.8)	1.000
Cerebrovascular Disease (%)	29 (9.3)	22 (9.4)	7 (9.0)	1.000
Arterial Hypertension (%)	140 (45.0)	112 (48.1)	28 (35.9)	0.067
Liver Disease (%)	23 (7.4)	17 (7.3)	6 (7.7)	1.000
COPD (%)	103 (33.1)	75 (32.2)	28 (35.9)	0.579
Emphysema (%)	97 (31.2)	71 (30.5)	26 (33.3)	0.673
Diabetes Mellitus (%)	40 (12.9)	32 (13.7)	8 (10.3)	0.558
Location of Tumour (%)				0.268
Central	68 (21.9)	55 (23.6)	13 (16.7	
Peripheral	243 (78.1)	178 (76.4)	65 (83.3)	
Tumour Diameter in mm (range)	21.50 (5.0–62.0)	22.35 (5.0–62.0)	18.95 (8.0–53.0)	**0.005**
UICC Stage (%)				0.313
IA	254 (81.7)	187 (80.3)	67 (85.9)	
IB	57 (18.3)	46 (19.7)	11 (14.1)	

aCCI: age-adjusted Charlson comorbidity index; BMI: body mass index; COPD: chronic obstructive pulmonary disease; FEV1%: forced expiratory volume in one second in %; MAT: mediastinal adipose tissue; ppoFEV1%: predicted postoperative forced expiratory volume in one second in %; SMCA: skeletal muscle cross-sectional area; SMI: skeletal muscle index; UICC: Union for International Cancer Control.

**Table 2:** Binomial logistic regression of risk factors for postoperative morbidity.

Variables in Equation								
	B	S.E.	Wald	df	Sig.	Exp(B)	95% C.I. for EXP(B)	
							Lower	Upper
Sarcopenia	0.912	0.382	5.699	1	0.017	2.489	1.177	5.261
Age	0.016	0.020	0.653	1	0.419	1.016	0.978	1.056
Sex	−0.394	0.388	1.031	1	0.310	0.674	0.315	1.443
ppoFEV1%	−0.043	0.015	8.031	1	0.005	0.958	0.931	0.987
Conversion to Thoracotomy	0.758	0.957	0.627	1	0.429	2.134	0.327	13.931
Extended Resection	0.882	0.721	1.497	1	0.221	2.415	0.588	9.912
Coronary Artery Disease	−1.013	1.082	0.875	1	0.349	0.363	0.044	3.030
Cerebrovascular Disease	−0.179	0.655	0.075	1	0.785	0.836	0.231	3.021
Chronic Kidney Disease	−0.503	0.845	0.354	1	0.552	0.605	0.115	3.170
Constant	−0.462	1.538	0.090	1	0.764	0.630		

ppoFEV1%: predicted postoperative forced expiratory volume in one second in %.

**Table 3:** Cox regression of risk factors for five-year overall survival.

Variables in Equation								
	B	SE	Wald	df	Sig.	Exp(B)	95% CI for Exp(B)	
							Lower	Upper
Sarcopenia	0.624	0.329	3.606	1	0.058	1.867	0.980	3.555
Age	0.026	0.015	2.827	1	0.093	1.026	0.996	1.058
Sex	0.265	0.302	0.772	1	0.380	1.304	0.721	2.356
ppoFEV1%	−0.012	0.011	1.329	1	0.249	0.988	0.967	1.009
Conversion to Thoracotomy	−12.376	397.674	0.001	1	0.975	0.000	0.000	.
Extended Resection	0.941	0.482	3.803	1	0.051	2.562	0.995	6.595
Coronary Artery Disease	0.898	0.621	2.091	1	0.148	2.454	0.727	8.288
Cerebrovascular Disease	0.399	0.484	0.680	1	0.409	1.491	0.577	3.849
BMI	−0.019	0.036	0.276	1	0.599	0.981	0.914	1.053

BMI: body mass index; ppoFEV1%: predicted postoperative forced expiratory volume in one second in %.

**Table 4:** Patient characteristics of the mediastinal adipose tissue subanalysis.

Factor	Total (n = 311)	Low/Medium MAT (n = 257)	High MAT (n = 54)	*p*-Value
Mean Age in years (range)	64.66 (15–83)	63.83 (15–83)	68.61 (51–80)	**<0.001**
Sex (%)				0.881
Female	149 (47.9)	124 (48.2)	25 (46.3)	
Male	162 (52.1)	133 (51.8)	29 (53.7)	
Mean BMI (range)	25.5 (14.1–42.3)	24.42 (14.1–42.3)	30.68 (23.7–39.8)	**<0.001**
Mean Height in cm (range)	170 (145–196)	169.57 (147–190)	170.17 (145–196)	0.663
Mean Weight in kg (range)	73.63 (38.0–118.0)	70.42 (38–102)	88.88 (65–118)	**<0.001**
Mean aCCI (range)	3.13 (0–8)	3.05 (0–8)	3.46 (1–6)	0.092
Mean SMCA in cm^2^ (range)	131.49 (72.93–199.26)	129.27 (72.93–199.26)	142.02 (87.05–194.24)	**0.005**
Mean SMI in cm^2^/m^2^ (range)	45.52 (26.43–70.22)	44.80 (26.43–70.22)	48.94 (32.11–68.82)	**0.002**
Mean FEV1% (range)	82.52 (34.0–154.8)	82.34 (34.0–154.8)	83.38 (48.0–118.0)	0.677
Mean ppoFEV1% (range)	65.83 (32.21–130.36)	65.22 (32.21–130.36)	66.16 (37.89–89.83)	0.656
Postoperative Complications (%)				0.225
No Complication	161 (51.8)	132 (51.4)	29 (53.7)	
Minor Complication	111 (35.7)	89 (34.6)	22 (40.7)	
Major Complication	39 (12.5)	36 (14.0)	3 (5.6)	
Sacropenia group (%)				0.124
No Sarcopenia	233 (74.9)	188 (73.2)	45 (83.3)	
Sarcopenia	78 (25.1)	69 (26.8)	9 (16.7)	
Coronary Artery Disease (%)	14 (4.5)	11 (4.3)	3 (5.6)	0.717
Cerebrovascular Disease (%)	29 (9.3)	25 (9.7)	4 (7.4)	0.798
Arterial Hypertension (%)	140 (45.0)	109 (42.4)	31 (57.4)	0.051
Liver Disease (%)	23 (7.4)	16 (6.2)	7 (13.0)	0.092
COPD (%)	103 (33.1)	96 (37.4)	7 (13.0)	**<0.001**
Emphysema (%)	97 (31.2)	84 (32.7)	13 (24.1)	0.259
Diabetes Mellitus (%)	40 (12.9)	26 (10.1)	14 (25.9)	**0.003**
Location of Tumour (%)				0.469
Central	68 (21.9)	54 (21.0)	14 (25.9)	
Peripheral	243 (78.1)	203 (79.0)	40 (74.1)	
Tumour Diameter in mm (range)	21.50 (5.0–62.0)	21.35 (5.0–62.0)	22.17 (8.0–47.0)	0.594
UICC Stage (%)				0.564
IA	254 (81.7)	208 (80.9)	46 (85.2)	
IB	57 (18.3)	49 (19.1)	8 (14.8)	

aCCI: age-adjusted Charlson comorbidity index; BMI: body mass index; COPD: chronic obstructive pulmonary disease; FEV1%: forced expiratory volume in one second in %; MAT: mediastinal adipose tissue; ppoFEV1%: predicted postoperative forced expiratory volume in one second in %; SMCA: skeletal muscle cross-sectional area; SMI: skeletal muscle index; UICC: Union for International Cancer Control.

## Data Availability

The data presented in this study are available on request from the corresponding author. The data are not publicly available due to institutional privacy policies.
